# Late Side Effects in Normal Mouse Brain Tissue After Proton Irradiation

**DOI:** 10.3389/fonc.2020.598360

**Published:** 2021-01-11

**Authors:** Theresa Suckert, Elke Beyreuther, Johannes Müller, Behnam Azadegan, Matthias Meinhardt, Felix Raschke, Elisabeth Bodenstein, Cläre von Neubeck, Armin Lühr, Mechthild Krause, Antje Dietrich

**Affiliations:** ^1^ German Cancer Consortium (DKTK), Partner Site Dresden, and German Cancer Research Center (DKFZ), Heidelberg, Germany; ^2^ OncoRay - National Center for Radiation Research in Oncology, Faculty of Medicine and University Hospital Carl Gustav Carus, Technische Universität Dresden, Helmholtz-Zentrum Dresden-Rossendorf, Dresden, Germany; ^3^ Helmholtz-Zentrum Dresden-Rossendorf, Institute of Radiation Physics, Dresden, Germany; ^4^ Helmholtz-Zentrum Dresden-Rossendorf, Institute of Radiooncology - OncoRay, Dresden, Germany; ^5^ Department of Physics, Hakim Sabzevari University, Sabzevar, Iran; ^6^ Neuropathology, Institute of Pathology, University Hospital Carl Gustav Carus, TU Dresden, Dresden, Germany; ^7^ Department of Particle Therapy, University Hospital Essen, University of Duisburg-Essen, Essen, Germany; ^8^ Department of Medical Physics and Radiotherapy, Faculty of Physics, TU Dortmund University, Dortmund, Germany; ^9^ National Center for Tumor Diseases (NCT), Partner Site Dresden, Dresden, Germany; ^10^ Department of Radiotherapy and Radiation Oncology, Faculty of Medicine and University Hospital Carl Gustav Carus, Technische Universität Dresden, Dresden, Germany

**Keywords:** proton therapy, brain irradiation, preclinical mouse model, magnetic resonance imaging (MRI), late side effects, blood–brain barrier (BBB), brain tissue toxicity, radiation dose modeling

## Abstract

Radiation-induced late side effects such as cognitive decline and normal tissue complications can severely affect quality of life and outcome in long-term survivors of brain tumors. Proton therapy offers a favorable depth-dose deposition with the potential to spare tumor-surrounding normal tissue, thus potentially reducing such side effects. In this study, we describe a preclinical model to reveal underlying biological mechanisms caused by precise high-dose proton irradiation of a brain subvolume. We studied the dose- and time-dependent radiation response of mouse brain tissue, using a high-precision image-guided proton irradiation setup for small animals established at the University Proton Therapy Dresden (UPTD). The right hippocampal area of ten C57BL/6 and ten C3H/He mice was irradiated. Both strains contained four groups (n_irradiated_ = 3, n_control_ = 1) treated with increasing doses (0 Gy, 45 Gy, 65 Gy or 85 Gy and 0 Gy, 40 Gy, 60 Gy or 80 Gy, respectively). Follow-up examinations were performed for up to six months, including longitudinal monitoring of general health status and regular contrast-enhanced magnetic resonance imaging (MRI) of mouse brains. These findings were related to comprehensive histological analysis. In all mice of the highest dose group, first symptoms of blood-brain barrier (BBB) damage appeared one week after irradiation, while a dose-dependent delay in onset was observed for lower doses. MRI contrast agent leakage occurred in the irradiated brain areas and was progressive in the higher dose groups. Mouse health status and survival corresponded to the extent of contrast agent leakage. Histological analysis revealed tissue changes such as vessel abnormalities, gliosis, and granule cell dispersion, which also partly affected the non-irradiated contralateral hippocampus in the higher dose groups. All observed effects depended strongly on the prescribed radiation dose and the outcome, i.e. survival, image changes, and tissue alterations, were very consistent within an experimental dose cohort. The derived dose–response model will determine endpoint-specific dose levels for future experiments and may support generating clinical hypotheses on brain toxicity after proton therapy.

## Introduction

Tumors of the central nervous system are still an entity with a very poor prognosis, with a current relative 5-year survival rate of around 19 – 22 % (1). However, patients of younger age (1) as well as those treated for low grade tumors (2) have a substantially better perspective. Preserving cognitive abilities and quality of life is of paramount importance to these patients. For radiotherapy, this requires the reduction of dose delivered to the tumor-surrounding normal tissue below a critical threshold dose.

An advantage of proton therapy (PT) over photon radiotherapy is its inherent physical properties: Particles stop in the tissue after depositing their energy maximum (Bragg Peak), leading to a reduced integral dose (3) and sparing of normal tissue. Thus, brain tumors and pediatric patients are often treated with this modality (4, 5). In recent years, there has been a surge in PT treatment facilities (6), and several smaller cohort studies indicate beneficial effects of PT such as improved overall survival (7) or prevention of brain-volume loss (8). However, data from randomized multi-center clinical trials is still lacking (9).

At the same time, preclinical data and observations from clinical practice call for a better biological understanding of the normal brain tissue toxicities after PT (10). While a constant relative biological effectiveness (RBE) of 1.1 relative to photon radiotherapy is used for treatment planning, several *in vitro* (11) and rare *in vivo* (12) studies suggest a variable and higher RBE. Increased RBE values occur particularly at the field edges, which are usually located in the normal tissue due to clinical safety margins. Additionally, particular brain areas such as the periventricular region (13, 14), the neural stem cell compartment (15, 16), and the corpus callosum (16, 17) are suspected to be more sensitive to radiation. If the higher RBE and the particular radiosensitivity of brain substructures prove to be clinically relevant, treatment planning and dose calculations would have to be adjusted accordingly (18).

Normal tissue toxicities caused by radiotherapy alone are hard to estimate in patients who often receive a combination of surgery, chemo- or immunotherapy (19) and also suffer from residual tumor, tumor recurrence or pseudo progression (20). Nevertheless, radiation-induced brain injury or neurologic complications are known side effects (4, 21). A suitable preclinical model would offer the potential to investigate radiation effects without confounding factors, model accurate predictions, and test effective counteractive measurements. Another advantage of the preclinical setting is the availability of tissue histology, which provides valuable insights into the underlying cellular changes.

Despite recent successes, meaningful *in vitro* models for normal brain tissue still fall short, with extensive cultivation requirements and missing complexity (22, 23). Classical *in vivo* brain irradiation experiments in rodents are performed with photons and designed to treat either the whole or half of the brain (24–27), which is not reflecting the clinical practice, where the irradiated normal tissue is minimized as much as possible. Since there is a strong dose-volume effect of normal tissue toxicities (28), more clinically relevant treatment fields are needed.

We recently established a workflow for high-precision proton irradiation of mouse brains (29, 30) and showed immediate DNA damage induction in a defined subvolume (31). Being able to reproduce clinical fields including a dose gradient and tissue sparing, investigating underlying tissue damaging mechanisms or alternative treatment options is now possible. As radiosensitivity differs not only between humans and mice, but also between mouse strains (32), it is mandatory to define the proton radiation doses that evoke side effects in murine brains comparable to clinical observation in patients. Therefore, the primary endpoints of this pilot study were (i) to determine a dose able to evoke clinically relevant tissue changes, and (ii) to elucidate the time dynamics of these side effects. To avoid age as confounding factor and enable experimental insights within a reasonable time frame, we decided to irradiate mice older than 60 days (33) and use a follow-up period of 6 month.

So far, no experimental data on high-dose proton irradiation of mouse brain subvolumes has been published, thus we relied on photon data of rat brain irradiation as reference point. The dose–response curve for the appearance of necrosis in irradiated rat brains lays between 20 Gy to 80 Gy at 19 month post irradiation (34) and image changes in MRI were observed for doses >30 Gy at 15 month after treatment (35). In the present study, we explored late side effects by applying increasing proton doses in a range of 40 Gy to 85 Gy. The longitudinal follow-up consisted of regular contrast-enhanced (CE) MRI, recording of animal health status, and final histological analysis. To model inter-patient variability, the two mouse strains C57BL/6 and C3H/He were compared as representatives for high radiation resistance and sensitivity (27, 32), respectively. In this way, we comprehensively characterized and established two robust, predictable animal models to tackle future research questions in the field of proton radiobiology.

## Material and Methods

### Animals

Eight to nine weeks old C57BL/6JRj (“C57BL/6”) and C3H/HeNRj (“C3H/He”) were delivered from Janvier Labs (Saint Berthevin Cedex, France) at least one week before starting the experiments. Only female mice were used to exclude potential sex differences. Animals were housed in Euro Standard Type III with up to five animals per cage at a 12:12 h light-dark cycle. Food, water, and Kaolin pellets (K50001, Brogaarden, Lynge, Denmark; “Pica test”) were available *ad libitum*. Nesting material as well as polycarbonate tunnels or mouse igloos were offered as cage enrichment. All experiments were approved by the Saxon authorities (Landesdirektion Sachsen, DD24.1-5131/449/32) and are in accordance with institutional, national, and European (EU Directive 2010/63/EU) animal welfare regulations.

### Magnetic Resonance Image Acquisition

MR images were acquired with a 1.0 T small animal MR scanner (nanoScan^®^ PET/MRI, Mediso Medical Imaging Systems, Budapest, Hungary) using a mouse head coil. Mice were anesthetized with 1–2 % isoflurane (Baxter Deutschland GmbH Medication Delivery, Unterschleißheim, Germany; vol./oxygen). The eyes were protected with Bepanthen eye cream (Bayer Vital GmbH, Leverkusen, Germany).

A field of view (FOV) covering the mouse brain was defined. In each imaging session, first a T2-weighted (T2w) MR scan was acquired with a 3-dimensional (3D) fast spin echo sequence (transverse slices, repetition time (TR) = 1,000 ms, effective echo time (TE) = 97.7 ms, FOV = 31.3 mm, 128 × 128 matrix size, slice thickness (ST) = 0.23 mm, number of slices (NS) = 90). Afterwards, Magnevist^®^ contrast agent (Bayer Vital, Leverkusen, Germany, ~ 5 µl/g body weight) was injected intraperitoneally (i.p.) 10 min before running a CE 3D gradient echo spoiled T1-weighted (T1w) sequence (transverse slices, TR = 15 ms, TE = 3.1 ms, FA = 25°, FOV = 60 mm, 256 × 256 matrix size, ST = 0.23 mm, NS = 90).

MR scans were recorded in the week prior to irradiation and biweekly thereafter, starting either in the first or second week after treatment. Measurements of the sham-irradiated animals as well as selected internal control scans, i.e. scans before irradiation, can be found in **Supplementary Figure S1**. After three months, the measurement interval was increased to up to five weeks. An additional diagnostic MR scan was acquired in case of decreasing health status of an animal.

### Irradiation Setup and Dosimetry

**Figure 1:**
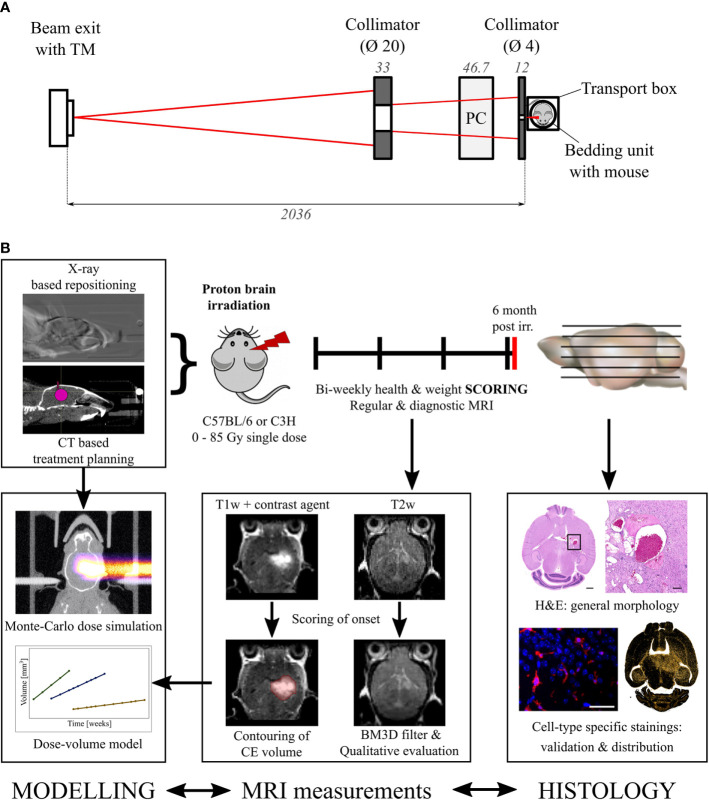
Irradiation setup and experimental procedure. **(A)** Schematic representation of the beam shaping system. Left to right: A 90 MeV proton beam (red) exits the vacuum beam line and passes through a transmission monitor ionization chamber (TM). A brass (left) and an aluminum collimator (right) shape the beam which is range adjusted by a polycarbonate (PC) compensator before irradiating the mouse within the transportation box. Dimensions in mm. **(B)** Overview of the workflow and the specified endpoints. Modelling, MRI measurements, and histology are correlated to find the dose evoking clinically comparable normal tissue toxicities.

**Figure 2:**
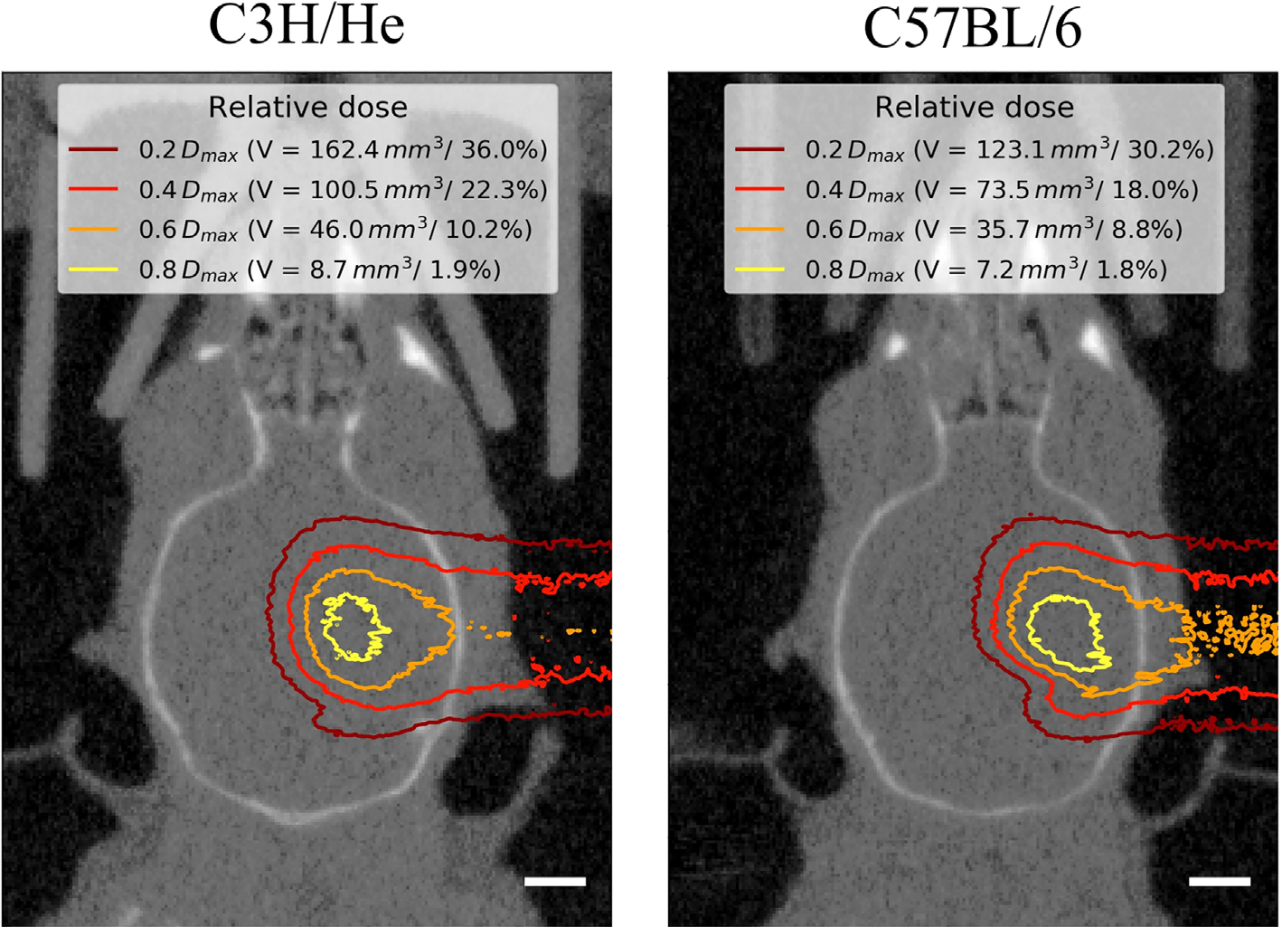
Representative mouse brain CTs in treatment position for one C3H/He and one C57BL/6 mouse with dose distributions from Monte-Carlo beam transport simulations. Little dose was deposited within the contralateral hemisphere. The extent of the brain that received at least a certain fraction of the dose maximum D_max_ is given as absolute volume V and as percentage of the brain volume. Scale bars 2 mm.

A 90 MeV proton beam was shaped laterally by an aluminum collimator with an aperture of 4 mm. To ensure that the Bragg peak position was in the mouse brain, the proton range was adjusted by a 47.6 mm polycarbonate range compensator. **Figure 1A** shows a schematic representation of the irradiation setup and **Figure 1B** an overview of the experimental design. The thickness of the range compensator was optimized using the Giraffe multilayer ionization chamber detector (IBA Dosimetry, Schwarzenbruck, Germany) to obtain a proton range in water of about 6.3 mm for the C3H/He mice. Because C57BL/6 mice are of smaller size, an additional 1 mm poly(methyl methacrylate) slab was added adjacent to the collimator, resulting in a proton range of about 4.9 mm in water (**Figure S2**). The 3D dose distribution, including the proton range at treatment position, was verified by EBT3 dosimetry film stacks [stopping power ratio 1.3 (36), Ashland Inc., USA, LOT: 04181701] that were calibrated beforehand with ionization chambers (capped Markus chamber, model 34045, PTW, Germany). Film readout was adapted for proton doses higher than 20 Gy through evaluation of the green color channel (37) using the fit procedures described by (38). Monte Carlo beam transport simulations of the 3D dose distribution in mouse brains and film stacks were performed by means of the software “Tool for Particle Simulation” [TOPAS (39),] as described in (31). **Figure 2** shows one representative simulated proton dose distribution for each mouse strain.

For absolute and relative dosimetry during mouse irradiation, a two-step process as described in (31) was applied. Briefly, the treatment dose was defined on the basis of EBT3 film stacks. The mean film dose within the 80% isodose area around the Bragg peak maximum was assumed as treatment dose. For comparison, a TOPAS simulation was performed to calculate the mean dose within the volume circumscribed by the 80% isodose line around the Bragg peak. Both, the dose estimated on basis of 2D films and the 3D simulated dose agreed within 10 %. In a second step, a correlation factor between treatment dose and mouse head entrance dose was determined as basis of the measured depth dose distributions. The entrance dose is easily accessible with EBT3 films placed at treatment position perpendicular to the incoming proton beam. To monitor the dose delivery during treatment, monitor units (MU) measured by the ionization chamber at beam exit (model 34058, PTW, Freiburg, Germany) were correlated to the mouse head entrance dose. Beam delivery was automatically switched off when the requested MU were reached. For mouse irradiation, a dose rate of about 10 Gy/min (Bragg peak) was applied to deliver physical doses (“high”, “intermediate”, “low”) of 80, 60, and 40 Gy to C3H/He mice, and of 85, 65, and 45 Gy to C57BL/6 mice. Each dose group contained three animals; one sham-irradiated animal per strain served as control. The quality assurance of dose delivery included the irradiation of EBT3 films with a defined dose range at entrance position to check for MU-dose correlation, and the irradiation of several film stacks during each campaign to verify the depth dose distribution.

### Animal Irradiation

At an age of 11–13 weeks, the right hippocampal area of the mice was irradiated with protons at the experimental beam line of the UPTD as described in (31). A designated mouse bedding unit (29) maintained mouse hygiene status and body temperature.

For treatment planning, a cone-beam computed tomography (CBCT) and orthogonal x-ray images were acquired in the week before radiation (40). We defined the target coordinates with the µRayStation 5 treatment planning software (RaySearch Laboratories, Stockholm, Sweden) using relative coordinates within the brain (cranial-caudal: 0.56, dorsal-ventral: 0.4) in accordance with the Allen Mouse Brain Atlas (41). On the treatment day, mice were anesthetized (i.p. ketamine (100 mg/kg, WDT eG, Garbsen, Germany)/xylazine (10 mg/kg, Serumwerk Bernburg AG, Bernburg, Germany)) and a second planar x-ray image was acquired. The two x-ray images were used to reposition the animal with the in-house developed RadiAIDD positioning software (https://github.com/jo-mueller/RadiAIDD).

### Scoring and Sample Processing

The health status of mice was scored twice per week on a scale from 0–5 considering reduction of body weight, behavior, general appearance, and the skin reaction (grade 0–4). The catalogue of scoring criteria was composed according to guidelines from (42) and (43) and a translation can be found in the Supplement (**Tables S1** and **S2**). To exclude bias and inter-observer variance, a majority of the scorings was performed by the same experienced observer, who was blinded for the applied radiation dose. Substitute observers were trained beforehand. Mice were removed from the experiment by cervical dislocation either when the score revealed deterioration of the animal’s health or after the maximum follow-up period of six month was reached. One C3H/He mouse of the 40 Gy group had to be censored in week 24 after irradiation due to a skin injury unrelated to the experiments. Brains were excised and fixed in 4 % formalin overnight at room temperature. Afterwards, tissue samples were processed for paraffin embedding with a semi-enclosed Benchtop Tissue Processor (Leica Biosystems, Wetzlar, Germany).

### Histochemistry

In brain areas with T1 contrast agent accumulation, consecutive paraffin sections in the transverse plane of 3 µm thickness were prepared every 100 µm and dried overnight at 37°C. Slices were dewaxed and rehydrated and—for immunohistochemistry—heat-induced antigen retrieval with citrate buffer (pH = 6) was conducted. Hematoxylin and eosin (H&E) staining was performed according to standard procedure.

For immunofluorescence, sections were blocked for 1 h at room temperature with 1x Rotiblock (Carl Roth, Karlsruhe, Germany, A151) supplemented with 0.1 % Triton X-100 (SERVA Electrophoresis GmbH, Heidelberg, Germany, 37240). Antibodies against glial fibrillary acidic protein (GFAP), ionized calcium-binding adapter molecule 1 (Iba1), Nestin, and the Ki-67 protein as well as their respective secondary antibodies were diluted in 1x Rotiblock and incubated either 1 h at room temperature or overnight at 4°C. After DNA counterstaining with 4′,6-diamidino-2-phenylindole (DAPI, Thermo Fisher Scientific, D3571), sections were embedded with fluorescence mounting medium (Agilent Technologies, Santa Clara, USA, S302380) and dried overnight. Antibody specifications as well as their respective concentrations are listed in **Supplementary Table S3**.

### Microscopy Image Acquisition and Analysis

Microscopic images were acquired with a 10x or 40x objective at a ZEISS Axio Scan.Z1 digital slide scanner (Carl Zeiss AG, Oberkochen, Germany) by the Light Microscopy Facility of the Center for Molecular and Cellular Bioengineering (CMCB) and with a 40x objective at an AxioImager M1 or Z2 (Carl Zeiss AG, Oberkochen, Germany). Images were post-processed using Zen 2.3 (blue edition, Carl Zeiss Microscopy GmbH, 2011) or, for visualization of gliosis, with Fiji (ImageJ 1.52p, 64 bit Windows) (44) by applying a background subtraction (rolling ball radius 20 pixel) and the look-up table “Red Hot”.

### Data Analysis and Statistical Evaluation

Appearing brain tissue changes were categorized by a neuropathologist (MM) in H&E samples. The volumes of regions with CE image signal on the T1w MR images were contoured by two independent experimenters each (**Figure S3**) using the Medical Imaging and Interaction Toolkit [MITK, v2018.04.2 (45)]. Total brain volumes were delineated in the T1w MR images by one observer for three mice per strain. T2w MRI data was filtered using BM3D (46) with a sigma of 1.5 (Matlab R2013a, The Mathworks Inc., Natick, MA, USA) for qualitative analysis, which comprised binary scoring of the presence of abnormal T2 signal intensities (hyper- and hypo-intense). The analysis was performed by three independent observers. GraphPad Prism 7 for Windows (Version 7.02, GraphPad Software, Inc., San Diego, CA, USA) was used for plotting Kaplan-Meier survival curves and survival analysis with a log-rank test. The group size of three animals in this pilot study circumvents any further statistical analysis.

### Dose-Volume Response Model

The onset time *t*_on_ of the first appearance of contrast agent accumulation in the T1w MR images after irradiation as well as the time evolution of the volume *V* were modelled as a function of irradiation dose *D*. The onset time,

(1) $$t_{on}(D)=t_{1}-t_{2}\ln\;(D/Gy),$$

was assumed to decrease logarithmically with increasing dose. The time constants *t*_1_ and *t*_2_ are model parameters and were obtained by matching Equation (1) to the experimental CE data for the C3H/He mice. The resulting model was then applied to the CE data of the C57BL/6 mice for model validation.

For follow-up time points *t* ≥ *t*_on_ (*D*), the logarithm of the CE volume,

(2) $$\log_{10}(V/ml)=a\;(D-D_{0})\;{\left(t/week\right)}^{b},$$

was assumed to increase linearly with dose and with follow-up time *t* to the power of *b*. The threshold dose *D*_0_ as well as the parameters *a* and *b* were obtained by globally fitting Equation (2) to the CE volume time series of all C3H/He mice. For the C57BL/6 mice data, the values for *a* and *D*_0_ obtained from the C3H/He data were maintained and only the parameter *b* was adapted to match the time dependence of the experimental outcome of all C57BL/6 mice. The model is included in the **Supplementary Excel File**.

## Results

**Figure 3:**
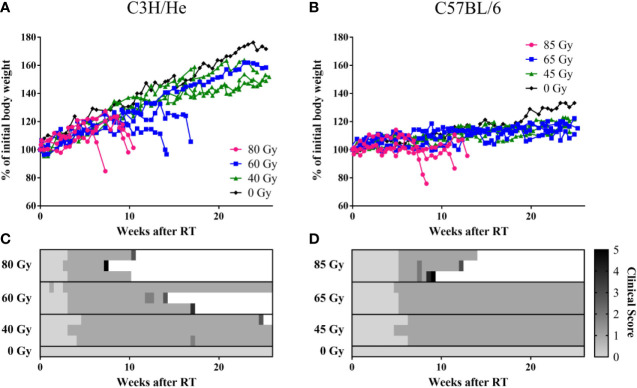
Weight curves of **(A)** C3H/He and **(B)** C57BL/6 shown as percent of initial body weight. **(C, D)** General health scoring of mice included skin reaction (grade 0–4), body weight reduction, general appearance, and behavior, according to a fixed set of criteria. A combined score of 5 was set as the stopping criterion.

In the weeks following treatment, general appearance and behavior of the mice remained normal and body weight either increased or remained stable (**Figures 3A, B**). All irradiated mice developed a skin reaction grade 1, i.e. dry desquamation and hair loss, approximately one month after treatment, with a slight delay in the lowest dose group (**Figure 3C**, clinical score change from 0 to 1). Effects appeared strictly within the irradiated area. The regrown fur lost its pigmentation, appearing white thereafter (**Figure S4**). Health deterioration occurred in C3H/He after high- (3/3) and intermediate- (2/3) dose irradiation and in C57BL/6 only in the highest dose group (3/3). If general well-being deteriorated, its decrease was rapid after a certain latency time and mice were sacrificed within few days according to the health scoring (**Figures 3C, D**). The major indicator of health decline was body weight loss; behavioral and appearance changes were recorded only on the last measurement day in most of the mice. Changes in body weight were more apparent in C3H/He animals. This is also reflected by their generally higher weight gain in the measurement period.

The Pica test as indicator for nausea showed no difference between irradiated and non-irradiated animals. Even when the animal suffered from radiation-induced weight loss, no clear indication of nausea, i.e. white colored feces, was observed. Autopsy after euthanasia always revealed an empty digestion tract, but no organ aberrations.

**Figure 4:**
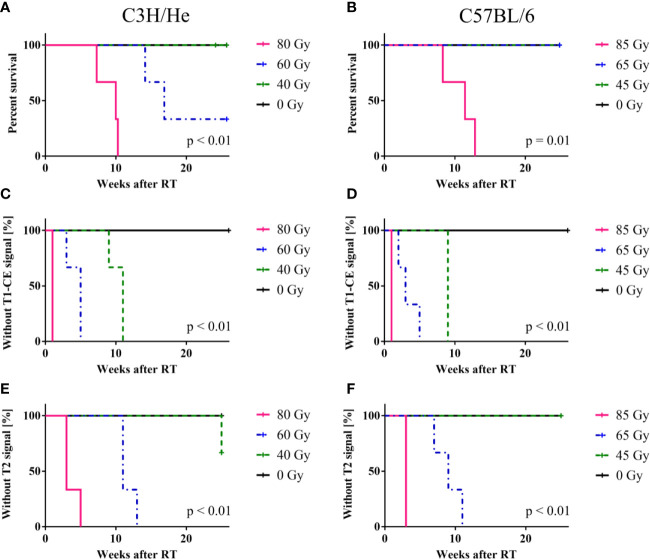
Mouse survival, onset of CE in T1w and signal onset in T2w MRI. **(A, B)** C57BL/6 mice showed an increased survival rate compared to C3H/He, whereas **(C–F)** onset times of MR image changes exhibited a similar pattern in both mouse strains, with a clear dose-dependency. T2w signal only appeared after T1w CE was observed. The log-rank test indicates that there is a significant difference in survival and signal onset in MRI between the dose groups.

We found prolonged survival for C57BL/6 mice compared to C3H/He mice (**Figures 3**, and **4A, B**) in the highest dose group [C57BL6: (76 ± 13) days, C3H/He: (64 ± 9) days]. All C57BL/6 mice irradiated with intermediate doses reached the maximal follow-up time, while only one out of three C3H/He mice in this dose group survived until the final measurement day. Animals irradiated with the lowest dose as well as the control animals survived until the end of the observation period.

**Figure 5:**
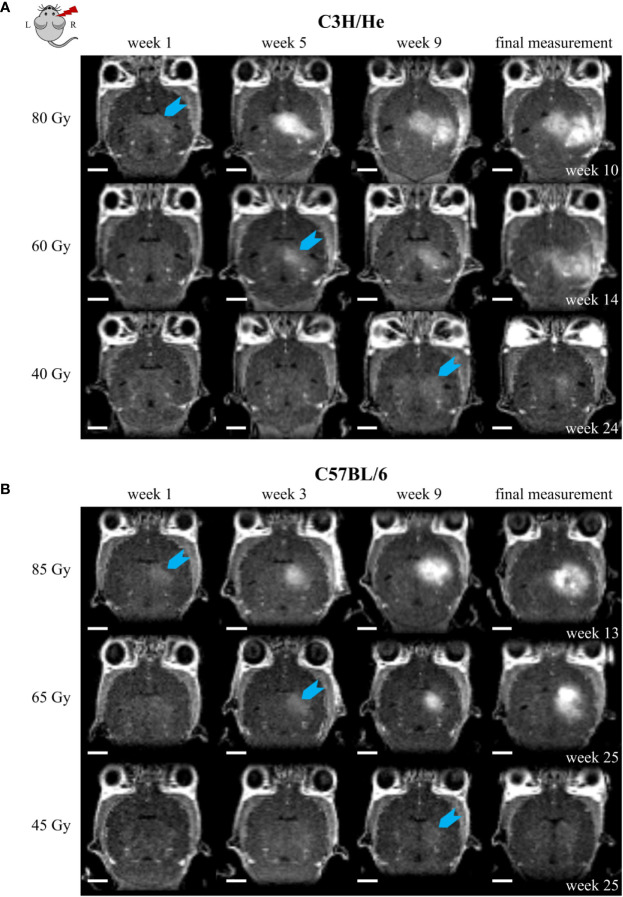
Exemplary CE T1w MR images of **(A)** C3H/He and **(B)** C57BL/6 for one selected mouse per dose group. The time points were chosen to reflect signal onset (blue arrow) in the different treatment groups. CE appeared sooner for higher doses. However, the progression pattern in the mouse strains matched and if the stopping criterion, i.e. health deterioration, was reached, affected volumes corresponded also between different doses. Scale bars 2 mm.

Regardless of the prolonged survival of C3H/He animals, MRI data showed contrast agent accumulation after similar dose-dependent onset times for both animal strains (**Figures 4C–F**). T1w CE appeared consistently before the occurrence of image changes in T2w MRI. We found no T2w hyper-intensities in animals exposed to 45 Gy, 40 Gy, and 0 Gy proton irradiation. Onset of CE was located within the area of the designated dose maximum (**Figures 2** and **5**).

**Figure 6:**
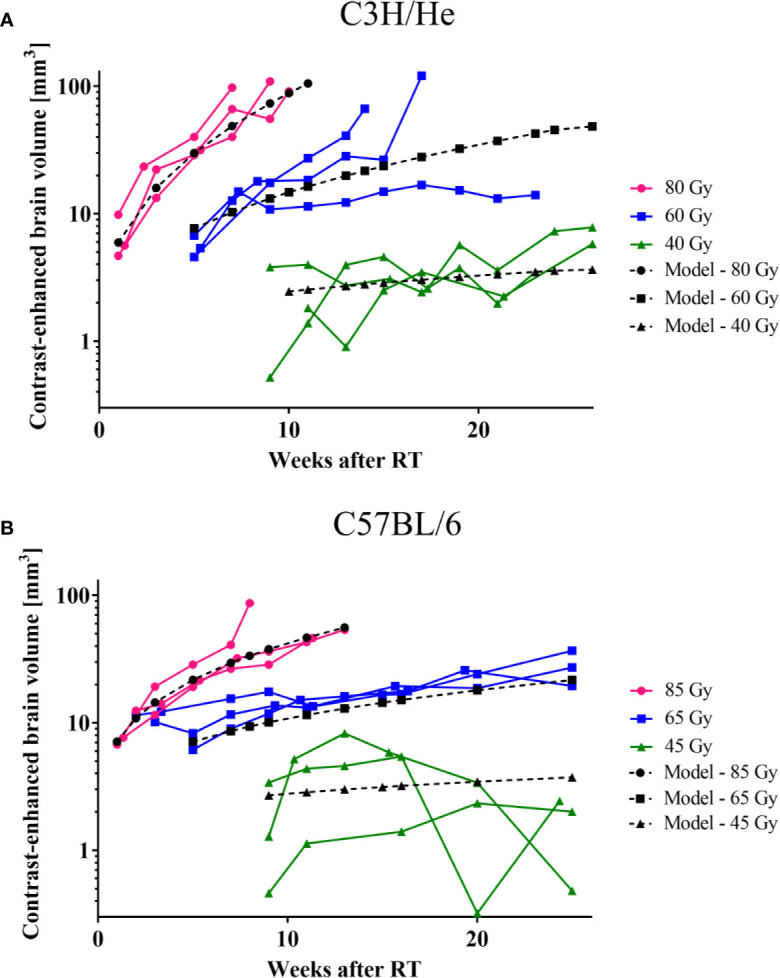
CE-volume increase in T1w MR images and the dose-volume response model (black curves) derived from experimental data for **(A)** C3H and **(B)** C57BL/6 mice. Onset and progression were earlier and faster for higher doses. C57BL/6 progressed at a lower rate than C3H/He animals.

The CE volume increased progressively after its initial occurrence (**Figure 6**). An exception was the lowest dose group (45 Gy) of C57BL/6 mice, where the data indicates an initial progression period followed by a mild recovery at the end of the observation period. The rate of CE volume growth strongly depended on the applied dose as well as on the mouse strain: Progression was faster for higher doses and C3H/He animals. To prevent implicit dependence of the observed effect on differences in total brain size between the two mouse strains, we contoured the total brain volume in three individuals and found comparable sizes (491.4 ± 16.7 mm^3^ and 488.6 ± 4.7 mm^3^ for C3H/He and C57BL/6, respectively). During the observation period, no noticeable brain volume increase occurred. While longer surviving animals showed slower progression, final CE volumes and health deterioration were within the same magnitude for all animals. In general, as expected for a normal tissue reaction, a high consistency within the dose groups was noted.

The acquired data was used to generate a dose-volume response model to predict the signal onset and the rate of CE volume increase based on Equations (1) and (2), respectively (**Table 1**). The onset time of the first image changes in MRI as function of dose showed for all six dose groups (i.e., both strains) a highly consistent dependence on dose and only small variances within each dose group. Onset time could therefore be precisely estimated by the according model for all 18 mice resulting in high R^2^ values of 0.97 and 0.92 for C3H/He and C57BL/6, respectively.

**Table 1:** Parameters for the dose-volume response model for irradiated brain subvolumes of C3H/He and C57BL/6 mice.

Mouse strain	*a* [Gy^−1^]	*b*	*D_0_* [Gy]	*t_1_* [weeks]	*t_2_* [weeks]
C3H/He	0.0155	0.40	30	55.75	−12.5
C57BL/6	0.0155	0.28	30	55.75	−12.5

For the modelling of the image change volume over time as a function of dose, in total 153 measured data points were included, that is, on average measured volumes at 8.5 time points were available per mouse. Accordingly, the time dependence of the image change volume was robustly modelled. On the other hand, only three different dose levels entered the modelling per mouse strain. To test model performance, first, a model was built on one cohort (all C3H/He mice, R^2^ = 0.80) and, second, applied to an independent cohort (all C57BL/6 mice, R^2^ = 0.76). Despite using two different mouse strains, only one model parameter (i.e., *b*) had to be adapted to fit the smaller CE volume growth rates observed for the C57BL/6 mice.

**Figure 7:**
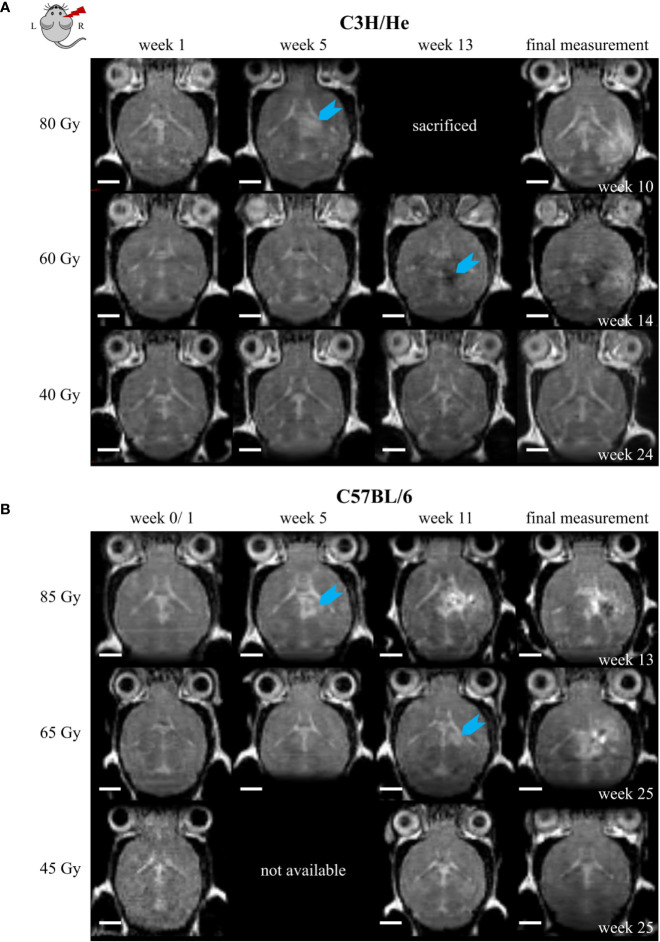
Exemplary T2w MR images of **(A)** C3H/He and **(B)** C57BL/6 for one selected mouse per dose group. Signal onset (blue arrow) occurred faster in high dose animals and did not appear in the lowest dose group. In most animals, the initial T2w image change was a hyper-intense signal; however, hypo-intensities were observed first in C3H/He mice irradiated with 60 Gy. Scale bars 2 mm.

T2w image changes followed the CE in T1w images with a time delay, but occurred initially within the same brain regions. T2w image changes comprised both T2 hyper- and hypo-intensities and generally had a more diffuse and heterogeneous appearance (**Figure 7**). However, at later time points the extent of T2 hyper-intense signal did outreach the volume with CE. T2 hyper-intensities were never observed in animals of the lowest dose group. On the other hand, one C3H/He animal irradiated with 40 Gy exhibited an area of hypo-intense T2w signal, which was also seen in the three C3H/He mice of the intermediate dose group before onset of hyper-intense signal. In C57BL/6, T2w hypo-intensities appeared as late effect for two animals (85 Gy and 65 Gy).

**Figure 8:**
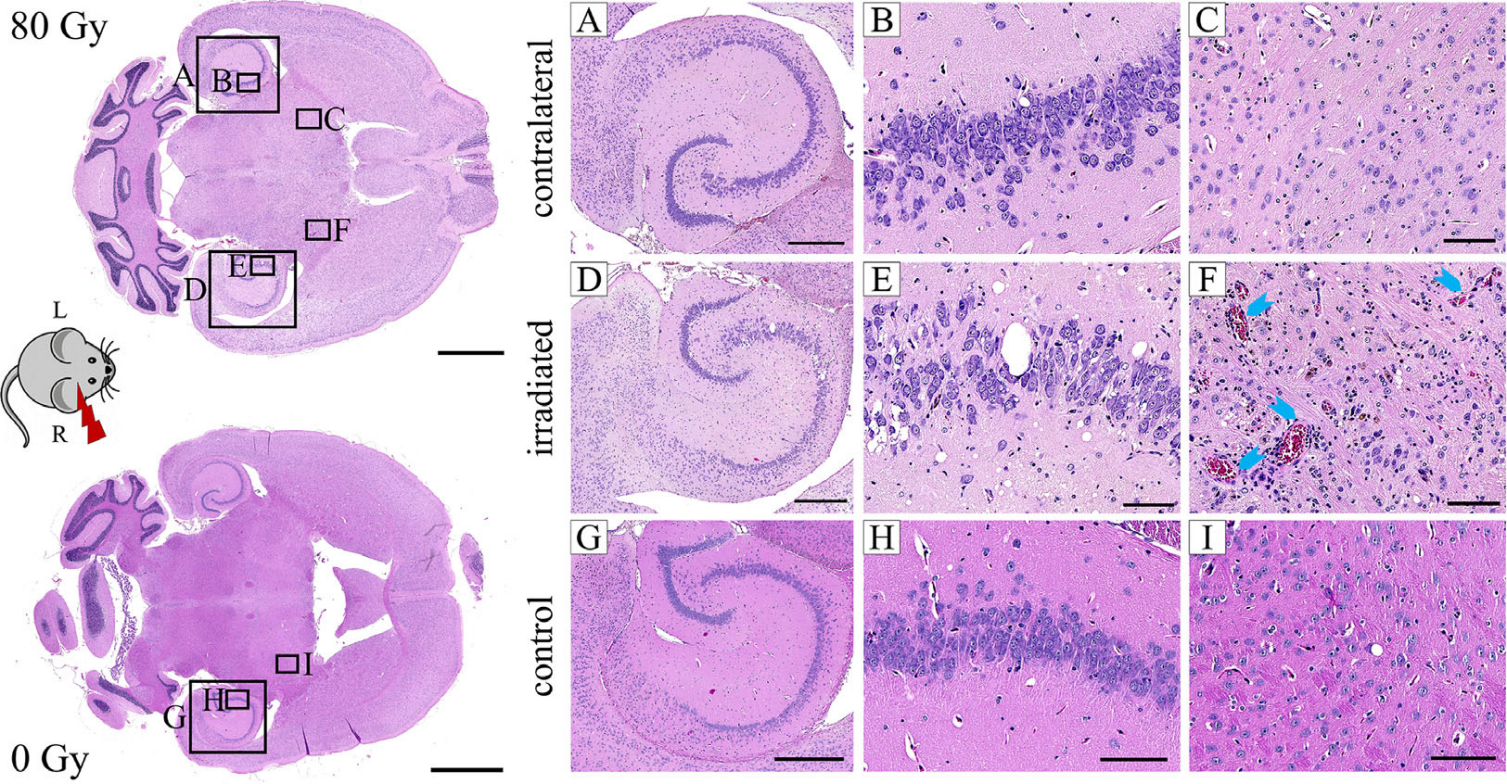
Exemplary H&E images of an 80 Gy proton irradiated **(A–F)** or control **(G–I)** C3H/He brain. The right hemisphere was exposed to proton radiation; the left hippocampus received no dose. Microfocal edema **(D–F)**, vessel proliferation **(D–F)**, hyalinosis and dilatation (**F**, blue arrows) of vessels, incomplete necrosis **(D–F)**, siderophages (**F**, brown color), and white matter damage **(D–F)** were noted in the irradiated brain. The hippocampus had a reduced cell density and granule cell dispersion **(D, E)**. None of these effects were present in the contralateral side **(A–C)** and the control animal **(G–I)**. Pink: parenchyma, violet: cell nuclei, red: erythrocytes, brown: siderophages. Scale bars (left to right): 2 mm, 500 µm, 100 µm, 100 µm.

Selected animals of each dose group were histologically evaluated to validate the MR imaging results and obtain pathologic findings on a microscopic level. H&E staining (**Figure 8**) revealed a broad range of normal tissue toxicities, which predominantly appeared within the irradiated field. We noted gross morphological alterations such as microfocal edema, white matter damage, and cytoplasmic changes. Fibrin extravasation, incomplete necrosis, and small areas of complete necrosis were present. Vessels had started to proliferate strongly and showed indications of dysfunction such as dilatation, chaotic organization, and hyalinosis. Gliosis of astrocytes and microglia occurred as well as microglia-lymphocyte nodules and macrophage invasion, mainly visible by residual siderophages, a sign of past micro-bleedings. Hippocampal sclerosis and granule cell dispersion were induced, especially in hippocampi irradiated with high doses. Incidence and severity of the side effects depended strongly on the delivered dose (**Table 2**). In general, the spatial transition from undamaged tissue to severe tissue alterations and vice versa was narrow and occurred within a range of 300 – 500 µm, which is attributed to the steep dose gradients of the proton beam. The H&E staining revealed only a weak effect in the low dose group.

**Table 2:** Overview of normal brain tissue toxicities observed after proton irradiation with different doses in C3H/He and C57BL/6.

		C3H/He				C57BL/6		
		40 Gy	60 Gy (a)	60 Gy (b)	80 Gy	45 Gy	65 Gy	85 Gy
**Morphology**	Complete necrosis							**x**
	Incomplete necrosis				**x**		**x**	**x**
	White matter damage		**x**	**x**	**x**		**x**	**x**
	Edema		**x**	**x**	**x**		**x**	**x**
	Fibrin extravasation						**x**	
	Subarachnoid hemorrhage						**x**	**x**
	Calcification	**x**	**x**					
**Vessels**	Proliferation	minor	**x**	**x**	**x**		**x**	**x**
	Hyalinosis		**x**	**x**	**x**		**x**	**x**
	Vasodilatation	minor	**x**	**x**	**x**		**x**	**x**
**Cellular changes**	Hippocampal granule cell dispersion		minor	**x**	**x**		minor	**x**
	Hippocampal sclerosis		minor	**x**	**x**		minor	**x**
	Gliosis		**x**	**x**	**x**		**x**	**x**
	Siderophages		minor		**x**		**x**	**x**

60 Gy (a): mouse survived 6 months observation period; 60 Gy (b): mouse died 17 weeks post irradiation.

**Figure 9:**
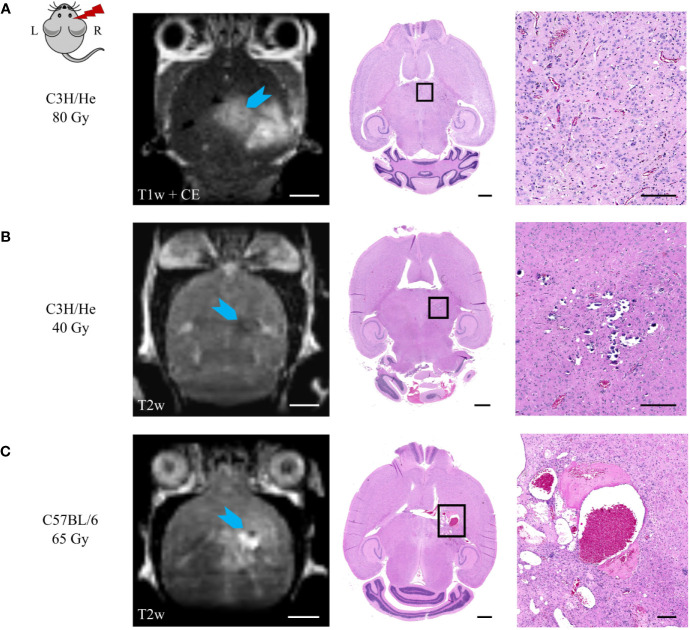
Correlation of MR image data and H&E histology. **(A)** Angiogenesis and vessel dilatation were observed in regions of contrast agent accumulation in T1w MRI. T2 hypo-intense signal could be related to **(B)** calcification or **(C)** fibrin extravasation and haemorrhage. **(C)** Hyper-intense spots in T2w sequences corresponded to edema and immensely dilated vessels. Scale bars (left to right): 2 mm, 1 mm, 200 µm.

Comparison of MRI data and histology could correlate image changes with tissue alterations (**Figure 9**). CE in T1w sequences appeared in regions with vessel proliferation and vasodilation. T2w hypo-intense spots were associated with calcification or haemorrhage and fibrin extravasation, whereas hyper-intense signal was linked to edema, angiogenesis, and vessel dilatation.

**Figure 10:**
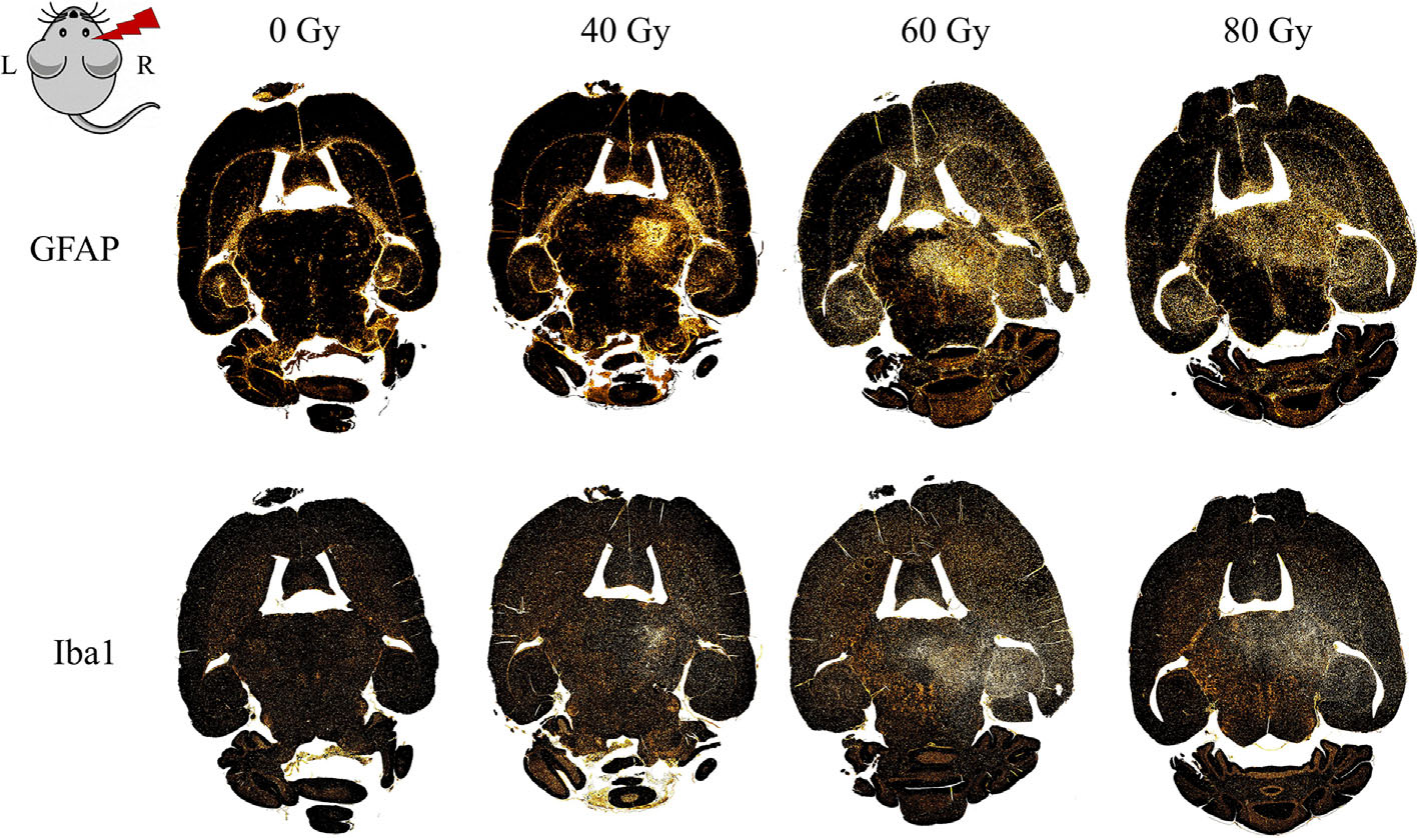
Distribution of gliosis in the proton irradiated C3H/He mouse brain. Gliosis is indicated by an increased number of astrocytes (GFAP, upper row) and microglia (Iba1, lower row) within the irradiated field and, for higher doses, also in the contralateral hippocampus. Staining was either performed within the same slice or in consecutive ones (within 15 µm distance, due to adaption of the staining protocol).

The microglial immune reaction, astrogliosis, and vessel aberrations were confirmed with further cell-type specific staining. This also revealed previously undetected effects in the low dose group, where abnormalities were not noticed in H&E histology. Proliferation of microglia (Iba1-positive) and astrocytes (GFAP-positive) occurred in the irradiation field, especially in the hippocampus, the area of the dose maximum, and around edema (**Figure 10**). Astrocytes formed a glial scar around injured tissue. At higher doses, there was—to a lesser extent—also an effect in the contralateral hippocampus with an increased GFAP expression. In general, tissue alterations were more pronounced and spatially widespread at higher doses.

**Figure 11:**
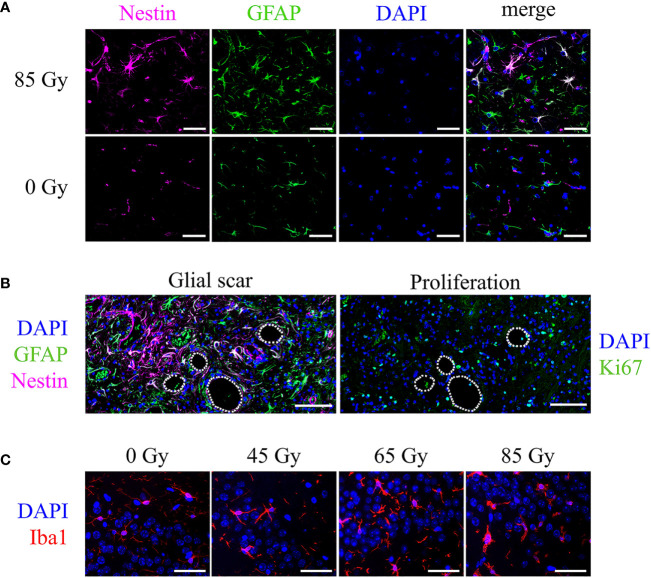
Cell type specific markers and cell nuclei (DAPI, blue) in representative irradiated brain sections of C57BL/6 mice. **(A)** Nestin-positive vessels (pink), GFAP-positive astrocytes (green), and Nestin-GFAP double-positive astrocytes in an animal irradiated with 85 Gy. No double-positive cells appear in the control animal. Scale bar 50 µm. **(B)** Consecutive slices showed the glial scar (left, Nestin-GFAP double-positive cells, green and pink) and proliferating, Ki-67-positive cells (right, green) located in the irradiated brain area, mainly around edema (white lines, 65 Gy). Scale bar 100 µm. **(C)** Iba1-positive microglia (red) increased in number and changed their morphology upon proton irradiation. Higher doses exhibit a bushy or amoeboid-like shape. Scale bar 50 µm.

The incidence of reactive gliosis was verified with a co-staining of GFAP and Nestin: double-positive cells, as well as a high number of Nestin-positive vessels were observed predominantly in the irradiated field and particularly within the glial scar tissue (**Figures 11A, B**). Brain areas affected by gliosis showed an increased amount of Ki-67 positive cells, indicating cell proliferation (**Figure 11B**). Brains of mice irradiated with 40 Gy/45 Gy formed no glial scar due to largely absent tissue damage and had only few double-positive cells as well as little reaction outside the irradiated area.

Microglia in the irradiated brain area increased in number and transformed their morphology to an activated state. Control animals show only resting local microglia with round, small cell somas and long, thin processes. Upon proton irradiation, cell shapes change from ramified to amoeboid with decreased cell spread, bushy processes, and increased soma sizes (**Figure 11C**). Again, changes were minor after low-dose (40 Gy/45 Gy) and more pronounced after high-dose (80 Gy/85 Gy) irradiation.

## Discussion

Normal tissue toxicity following radiotherapy is a clinical challenge, particularly in brain tumor patients, and the availability of meaningful preclinical models is crucial for understanding the induced side effects. Existing preclinical studies focus on half- (47) or whole-brain irradiation (27) and thereby disregard the dose-volume effect, impairing clinical relevance. In this pilot study, we established and comprehensively characterized a suitable *in vivo* model to investigate long-term side effects after proton irradiation of the hippocampal area as a relevant brain subvolume. For this, we used a follow-up with longitudinal evaluation of MR image changes and general well-being in addition to final histopathological findings.

The incidence, latency period, and severity of observed normal tissue toxicities, i.e. MR image changes, skin reaction, weight reduction, and histological abnormalities, strongly depended on the delivered dose for all investigated endpoints, as found previously (34, 35, 48). Interestingly, all observed effects were very consistent across the different dose groups although the experiments were planned as a pilot study with n = 3 mice per condition. As a first visible side effect, hair loss and subsequent whitening of the fur occurred. This can effectively be used to determine the correct application position of the proton beam. The Pica test for detection of nausea or headaches did not reveal any outcome and is thus not helpful to assess animal burden in this context. While apparent behavioral changes occurred late, body weight changes have proven as a reliable indicator for mouse health deterioration after proton brain irradiation. Therefore, a meaningful scoring system for future experiments should consider the weight kinetics. The investigated two mouse strains were chosen to represent a patient population with heterogeneous radiosensitivities (32); the proposedly more radiosensitive C3H/He showed a faster progression of therapy-related BBB damage and weight loss. However, our data may not allow a definite conclusion on that matter, since C3H/He had a larger fraction of their brain volume irradiated, leading to a higher integral dose. In particular the small dimensions of the preclinical setup combined with anatomic differences between the mouse strains complicate the irradiation of identical volumes. Alternatively, a voxel-wise correlation of dose and damage might be a promising approach to reveal strain-specific differences as well as variances in radiation response between the brain regions.

In the MRI measurements, the earliest detectable image change was contrast agent accumulation, which implies leakage into the tissue and signifies increased vessel permeability or breakdown of the BBB. CE in T1w MR images was observed in all irradiated animals and formed sharp outlines, thus enabling a clear differentiation from the surrounding brain tissue. On the other hand, T2w hyper-intensities occurred only in intermediate and high dose animals and exhibited a diffuse appearance. Retrospective analysis of H&E staining could verify the existence of white matter damage, edema, and incomplete necrosis in brain areas with CE and hyper-intense MR image signal. In two of the animals with T2w hypo-intensities, histology revealed calcification in the respective region, in another animal subarachnoid hemorrhage and fibrin extravasation were diagnosed. This is in agreement with existing literature: intracerebral bleeding, accumulation of mineral substances, or protein-containing lesions are some morphological changes causing T2w hypo-intensities in patients (49). Origin of the image changes in both sequences was consistently in the region of the dose maximum, but with progressing side effects, surrounding tissue was affected and a T1/T2 mismatch occurred. Above-mentioned MR image changes are classic clinical features of late radiation injury and frequently occurring in patients after brain radiotherapy (50–52) as well as in previous reported preclinical experiments (35, 47, 53, 54). The distinctive feature of our study is the in-depth comparison of MR image changes with tissue alterations in histology. One restriction was the rather low MRI signal-to-noise ratio combined with a weak T2-weighting of the sequence. This impaired more differentiated evaluation of imaging data and may provide an explanation for the onset of T2w image changes, which appeared consistently after T1-CE. Clinical data frequently describes an inverted course of events with an earlier occurrence of image changes in T2w imaging (52). However, contrast agent accumulation was clearly distinguishable and the onset and qualitative evaluation of image changes in T2w images was possible after applying a BM3D noise reduction filter. Hence, the imaging protocol was deemed feasible as it allowed for high-throughput measurements with acceptable scan times. From the MRI data, we could derive a dose-volume model that consistently predicts onset time and progression of the BBB breakdown as well as animal survival. This model can now be used to choose suitable dose levels for evoking clinically relevant radiation toxicities at realistic preclinical time points in the two mouse strains C3H/He and C57BL/6. Despite the small animal number, we consider the proposed model from this pilot study useful and applicable, due to its consistency and successful validation in an independent cohort. Especially the finding that increased radiation dose leads to quicker onset of radiation pathology confirms the outcome of earlier *in vivo* mouse photon irradiation studies (55). Additional testing of the model in future experiments with larger cohorts and especially at other dose values is recommended. Future studies could investigate to which extend the modelled behavior also applies to cancer patients suffering from radiation-induced tissue damage, where a similar progression of CE volume increase was observed. In particular, the observed distinct dose dependence of the onset time and velocity of CE volume increase should be tested in an appropriate clinical cohort. Taken together, our findings clearly prove the value of preclinical experiments for interpreting medical imaging results, in turn helping to improve patient diagnosis.

For a deeper understanding of the tissue changes, specific cell types were investigated in greater detail. The strong gliosis observed in H&E staining proved to be originated from both astrocytes and microglia. Ki67-positive cells were present in the respective area, demonstrating active local proliferation. Especially around injured tissue, such as edema or hemorrhages, Nestin-positive astrocytes appeared. These cells are known to form the so-called “glial scar”, which is a typical mechanism to self-limit tissue damage that protects against invading inflammatory cells (56). Microglia recruitment to the radiation lesion and cell activation was observed in all dose groups, but the extent showed a clear dose dependence visible in both cell distribution and morphology. Especially after high-dose irradiation, microglia were highly circular with big soma sizes, signifying activation (57). Interestingly, the contralateral hippocampus was affected by gliosis. This has been described before (58) and was attributed to global neuroinflammation resulting from brain irradiation (59). After severe trauma and BBB damage, additional invading immune cells take part in the inflammatory reaction. We did not observe peripheral macrophages after low dose irradiation, whereas specimen of higher doses showed residual siderophages, i.e. blood cell clearing macrophages as a sign of past micro-bleedings, and occasional lymphocyte-microglia nodules. Another prominently changed tissue component is the vasculature, exhibiting vessel dilatation, -hyalinosis, and -angiogenesis. These alterations have also been observed in patient biopsies or autopsies (60, 61) and indicate dysfunctional blood flow and ensuing limited nutrient supply in the respective tissue. The underlying reason could be radiation-induced microvascular injury and a subsequent decline of the vessel population, leading to tissue hypoxia. Apoptosis of CNS vessels as early as 24 h post irradiation has been observed, and experimental data indicates a slow recovery of endothelial cell density with leaky immature vessels (62, 63). Studies on sleep apnoea (64) and brain radiation (65) reveal angiogenesis, reactive gliosis, neuroinflammation, and altered hippocampal neurogenesis as reaction to hypoxic conditions. Vessel proliferation, inflammation, and reactive astrogliosis were indeed confirmed in our final histopathological analysis; and even though altered neurogenesis was not investigated, it is possibly one mechanism contributing to the observed massive gliosis (58). Two hitherto unidentified histological changes following radiation are granule cell dispersion and hippocampal sclerosis, which are known from epilepsy (66) or neurodegenerative diseases (67), but not radiotherapy. Since patient biopsies or autopsies of irradiated hippocampi are very rare, further preclinical studies will be needed to elucidate whether these side effects may contribute to neurocognitive decline after brain radiation.

Our results suggest that alleviating or reversing therapy-related toxicities might be possible; for example by reducing the inflammatory reaction (59) or protecting the vasculature by VEGF blockade (68, 69). The mouse model presents a valuable tool to screen for such potential treatment approaches and characterize their underlying mechanisms before entering clinical trials. Future studies should include longitudinal histology as well as high resolution T1w and T2w MRI together with additional sequences that represent the current clinical standard, such as FLAIR imaging and diffusion weighted imaging. This would allow for improving the link between MR image changes seen clinically after radiotherapy to preclinical MRI and, most importantly, the underlying histological changes. Additionally, high resolution MRI could help to elucidate radiation-induced normal tissue toxicities further, e.g. regarding specific brain regions of interest such as the periventricular area (13, 14) which appears to be particularly radiosensitive. Complementary “omics” analysis or liquid biopsies could reveal suitable biomarkers for predicting the potential onset or the occurrence of late side effects, which could support patient stratification and treatment. Furthermore, the influence of hippocampal-sparing proton irradiation on cognitive abilities, the role of the stem cell niche, and the beneficial potential of exercise during and/or after radiotherapy (70) are interesting research questions which can be tackled using the presented model. This pilot study could therefore achieve its primary endpoints: (i) we defined a dose delivered as a single fraction by a laterally confined 4 mm proton beam in one brain hemisphere within the range of 40 – 50 Gy as suitable for preclinical experiments focused on clinically relevant normal tissue alterations after brain radiotherapy. (ii) The chosen follow-up time of 6 month proved long enough to capture relevant tissue changes while still enabling data analysis within a reasonable time frame. The onset and dynamics of observed MR image changes provide valuable insight for deciding on appropriate analysis time points in longitudinal studies for maximizing the significance of the results while minimizing the number of animals needed.

In summary, we were able to induce normal tissue changes in murine brains similar to clinical observations following partial brain irradiation using a proton beam with a clinically relevant field formation. From MRI measurements, we established a dose–response model, which can be applied for more accurate experimental planning. In addition, histology identified hippocampal sclerosis and granule cell dispersion as potential side effect of radiation which could contribute to neurocognitive decline. With regard to future studies, our model offers the possibility to study existing and generate new clinical hypotheses for radiation-induced brain damage, reveal underlying mechanisms in greater detail, and, most important, find suitable treatment and prevention strategies.

## Data Availability Statement

Contoured MRI volumes and onset times are included in the article as supplementary material. Requests to access MRI raw data and the histology should be directed to the corresponding author.

## Ethics Statements

The animal study was reviewed and approved by the Saxon authorities (Landesdirektion Sachsen, DD24.1-5131/449/32).

## Author Contributions

TS, EBe, CV, AL, MK, and AD participated in the conception of the study. Data acquisition was performed by TS, EBe, AL, JM, MM, EBo, and AD. Analysis and interpretation of data were executed by TS, EBe, JM, BA, MM, FR, AL, MK, and AD. JM, FR, and TS provided the software. MK acquired funding. Project supervision was offered by EBe, MK, and AD. Visualization was done by TS and JM. The first draft of the manuscript was written by TS. AL and EBe wrote parts of the manuscript. All authors revised the manuscript and approved the final version

## Funding

Proton irradiation was done at the University Proton Therapy Dresden (UPTD) which has received funding from the European Union's Horizon 2020 Research and Innovation Programme under Grant Agreement No: 730983 (INSPIRE).

## Conflict of Interest

The authors declare that the research was conducted in the absence of any commercial or financial relationships that could be construed as a potential conflict of interest.
