# Supplementary material for: Late Side Effects in Normal Mouse Brain Tissue After Proton Irradiation
Source: Front Oncol. 2021 Jan 11;10:598360. doi: 10.3389/fonc.2020.598360 (PMC7842140; doi:10.3389/fonc.2020.598360)
Supplement: Supplementary file 1 [file DataSheet_1.pdf]

Supplementary

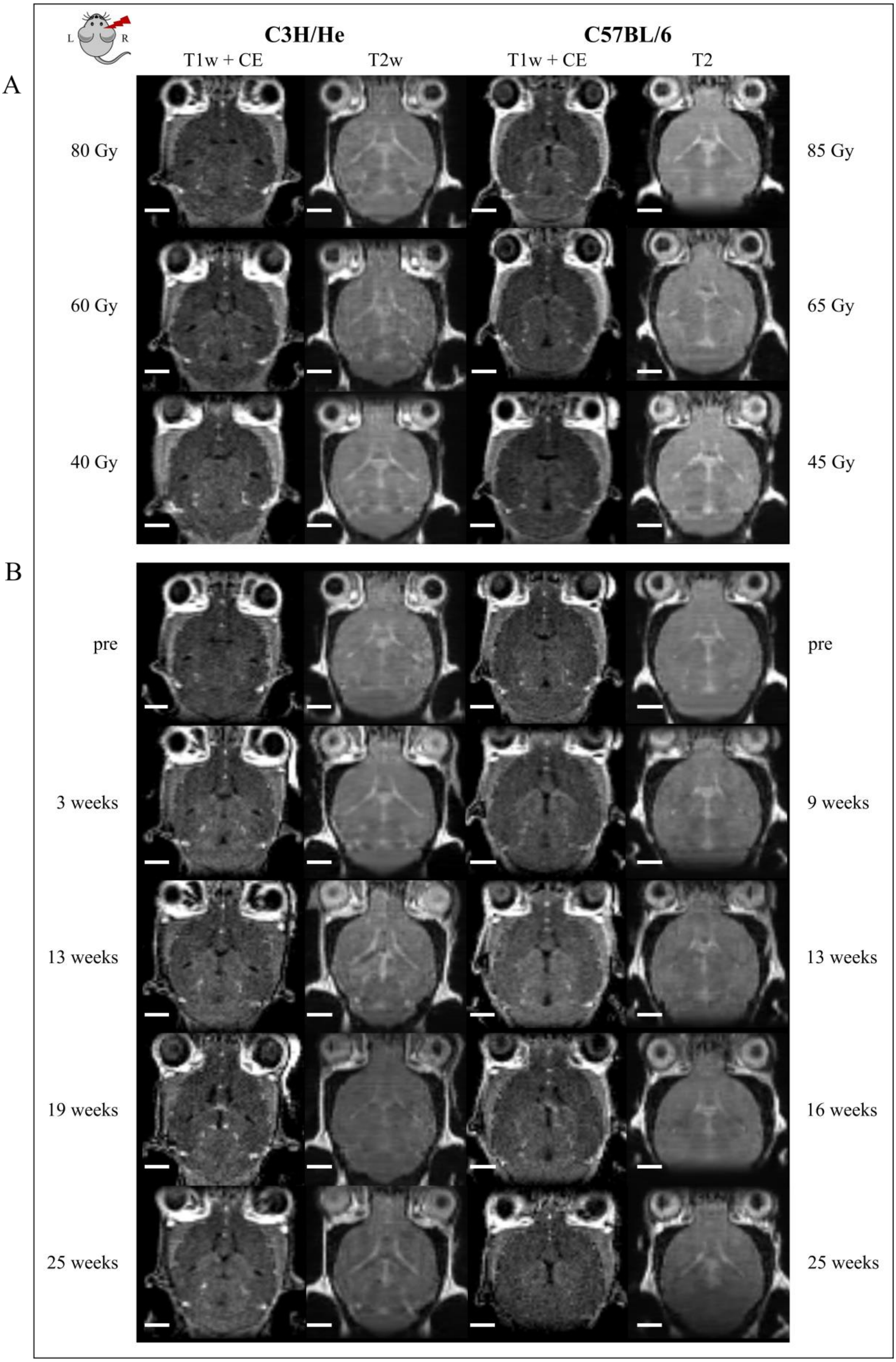

Figure S1: Control MRI measurements. (A) Scans before irradiation served as internal control for each individual mouse. One selected mouse per dose group and strain is shown as example. (B) Longitudinal measurements of the unirradiated age controls. No alterations were observed for any of the control MRI scans. Scale bars 2 mm.

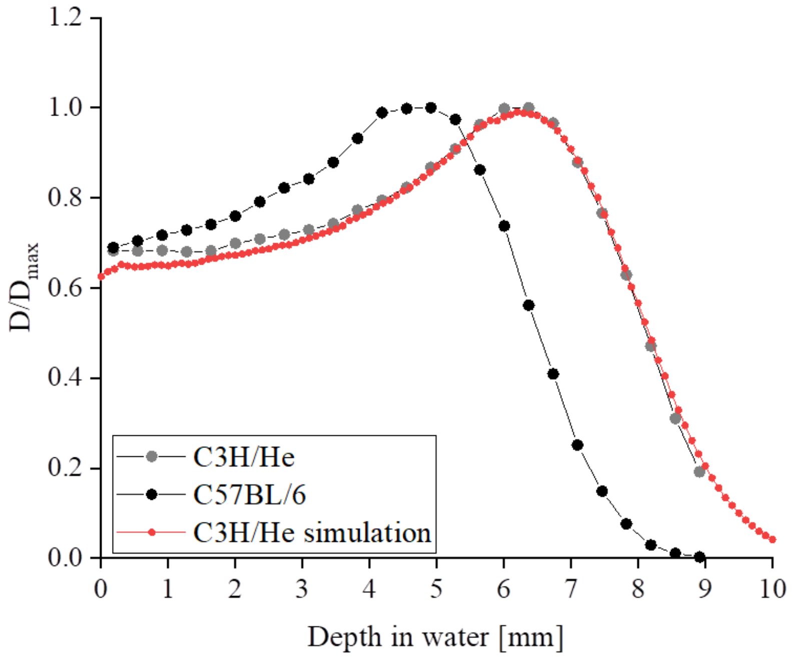

Figure S2: Proton depth dose distributions reconstructed from EBT3 film stacks at mouse head position in the respective setup for C57BL/6 (black dots) and C3H/He mice (grey dots). For comparison, the depth dose distribution simulated on basis of a computed tomography of an EBT3 film stack at the position of a C3H mouse is shown (red). The small deviation at the entrance is probably caused by positioning uncertainties of the film stack.

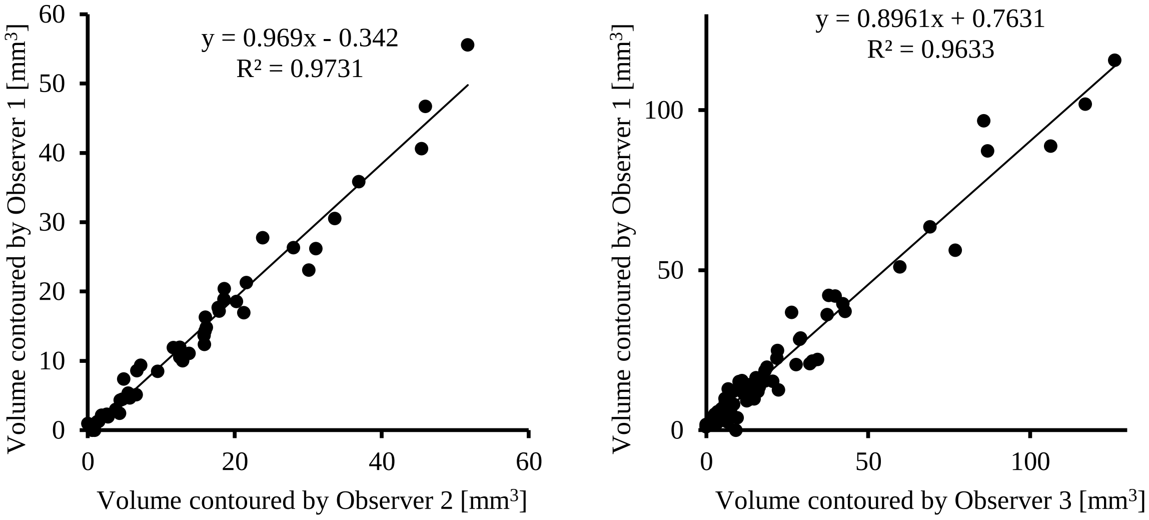

Figure S3: Inter-observer variability of CE volume contouring. Each measurement was evaluated by two independent experimenters, with comparable outcomes.

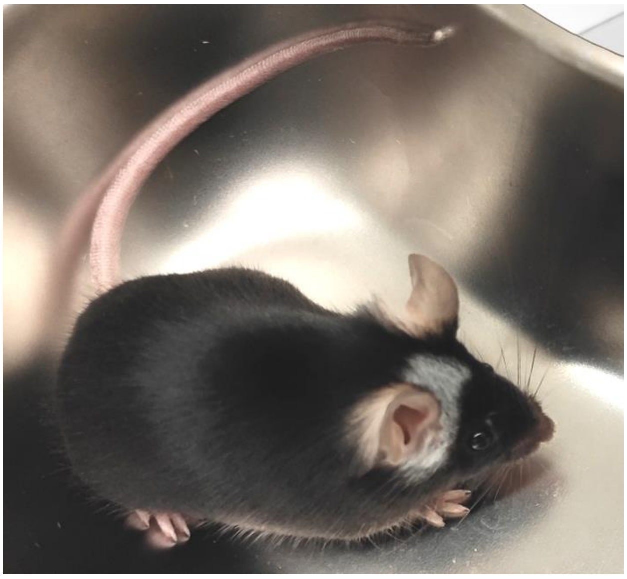

Figure S4: Hair whitening in a C57BL/6 mouse after proton brain irradiation of the right hemisphere.

Table S1: Score Sheet for mouse health assessment

Parameter	Observation	Score
Reduction of body weight	normal	0
	8-10% reduction	1
	10-15% reduction	2
	> 20% reduction	5
General appearance	open, clear eyes; even, normal breathing; clean orifices	0
	reduced or missing grooming; untended orifices	1
	clotted orifices, increased tonicity, visibly enhanced breathing	2
	bent, abnormal body posture; unclear or closed eyes; high tonicity; strongly enhanced breathing	5
Behaviour	normal; regular food and water intake; aktiv and aware, curious; social contacts, grooming	0
	slight alterations, decreased reaction, partly reduced mobility and motor functions	1
	unusual, crouching; partly apart from group; slight ataxia; reduced movements	2
	strongly reduced activity, defence and social behaviour; self-isolation, apathy, palsy; sounds of pain during handling	5
Skin reaction in and around the irradiated area	no acute skin reaction	0
	skin reaction grade 1	1
	skin reaction grade 2	2
	skin reaction > grade 2	5

**Score 1-2:**	minor burden; monitor closely
**Score 3-4:**	intermediate burden; inform veterinarian, analgesia if necessary; closer observation (every 1-2 days)
**Score 5 or more:**	high burden, immediately sacrifice animal by cervical dislocation

Table S2: Score Sheet for grading of skin reaction. Adapted from (43).

Grade	Observation
0	normal
1	follicular, faint or dull erythema; epilation; dry desquamation
2	tender or bright erythema; patchy moist desquamation; moderate edema
3	confluent, moist desquamation; pitting edema
4	ulceration; hemorrhage; necrosis

Table S3: List of used antibodies

#	**Primary antibodies**	**Supplier**	**Cat.no.**	**Dilution**	**2nd #**
1	rat anti-GFAP	EMD Millipore	345860	2000	1 or 2
2	rabbit anti-Iba1	Abcam	ab178846	1000	3 or 4
3	chicken anti-Nestin	GeneTex	GTX85457-50	1000	5
4	rat anti-Ki67	BD Bioscience	14-5698-80	200	1

#	**Secondary antibodies**	**Supplier**	**Cat.no.**	**Dilution**	**1st #**
1	anti-rat Alexa488	Invitrogen	A21208	500	1, 4
2	anti-rat Alexa594	Abcam	ab150152	500	1
3	anti-rabbit Alexa488	Invitrogen	A21206	500	2
4	anti-rabbit Alexa594	Invitrogen	A11012	500	2
5	anti-chicken Dylight750	Invitrogen	SA5-10075	500	3
